# Multi‐Band Excitation of NIR‐II Lanthanide Nanoparticles via Multiple‐Dye Cascade Sensitization

**DOI:** 10.1002/anie.2349085

**Published:** 2026-05-17

**Authors:** Yuxia Luo, Yunge Du, Yin Huang, Lijun Jiang, Chengyi Chu, Guochen Bao

**Affiliations:** ^1^ College of Bioresources Chemical and Materials Engineering Shaanxi University of Science & Technology Xi'an Shaanxi China; ^2^ School of Mathematical and Physical Sciences, Faculty of Science University of Technology Sydney Sydney New South Wales Australia; ^3^ Hubei Key Laboratory of Genetic Regulation and Integrative Biology Key Laboratory of Pesticide & Chemical Biology of Ministry of Education School of Life Sciences Central China Normal University Wuhan China; ^4^ Laboratory of Atomic‐Scale and Micro & Nano Manufacturing Ningbo Institute of Materials Technology and Engineering Chinese Academy of Sciences Ningbo China; ^5^ Institute for Biomedical Materials and Devices (IBMD) Faculty of Science University of Technology Sydney Sydney New South Wales Australia

**Keywords:** emission‐tuning, lanthanide‐doped downshifting nanoparticles, multi‐band excitation, multiple‐dye sensitization, NIR‐II luminescence

## Abstract

Lanthanide‐doped downshifting nanoparticles (DSNPs) emitting in the near‐infrared‐II (NIR‐II) window offer distinct advantages for deep‐tissue optical applications, yet their practical utility is fundamentally constrained by excitation limited to a few discrete wavelengths. Here, we report a multi‐band‐driven lanthanide nanoparticle platform enabled by a multiple‐dye sensitization strategy. By integrating three complementary organic dyes—tropolone (Trop), cyanine 3 (Cy3), and IR806—the excitation window of DSNPs is expanded sixfold, across the ultraviolet–visible–near‐infrared (UV–Vis–NIR) region from 270 nm to 1030 nm. While Trop and IR806 exhibit efficient sensitization, IR806 further acts as an energy‐transfer bridge, enabling efficient cascaded energy transfer and markedly enhanced NIR‐II emission for the Cy3 sensitization (efficiency increased from ∼6% to 70%). This modular strategy affords tunable NIR‐II emission from 1060 nm to 1525 nm, providing unprecedented flexibility in excitation and emission wavelength selection for advanced optical applications.

## Graphical abstract

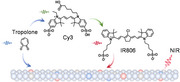

## 1 Introduction

Near‐infrared‐II (NIR‐II, 1000–1700 nm) irradiation has intrinsic advantages of low tissue autofluorescence, reduced photon scattering, and deep penetration depth, compared with visible and NIR‐I wavelengths. These advantages render NIR‐II light highly attractive for bioimaging and light‐assisted chemical transformations [1, 2, 3, 4, 5, 6]. Within this context, lanthanide‐doped downshifting nanoparticles (DSNPs) have emerged as a particularly attractive class of NIR‐II emitters, offering narrow emission bands, long luminescence lifetimes, high photostability, and low cytotoxicity. These unique characteristics make DSNPs promising candidates for applications ranging from deep‐tissue bioimaging [7, 8, 9, 10], multiplexed detection [11, 12, 13], and photo‐assisted therapies [14, 15, 16, 17] to information encryption [10, 18, 19] and environmental monitoring [20].

Significant progress has been made in enhancing the NIR‐II emission efficiency of DSNPs through a variety of strategies, including optimization of lanthanide dopant concentrations [21, 22, 23, 24, 25], core‐shell structural engineering, surface modification [26, 27, 28, 29, 30], promotion of phonon‐assisted cross relaxation pathways [31, 32, 33], reduction of crystal field symmetry [8, 34, 35], plasmonic modulations [29, 36, 37], and dye‐sensitization [38, 39, 40]. Despite these advances, the excitation of DSNPs remains largely restricted to a limited number of low‐energy wavelengths, most notably 808 nm for Nd^3+^‐sensitized systems and 980 nm for Yb^3+^‐sensitized systems. Although certain lanthanide ions such as Tb^3+^ and Eu^3+^ can in principle enable excitation in the visible region, practical extension into the visible and ultraviolet regions is severely restricted by the intrinsically narrow absorption bands of 4f–4f transitions and the limited availability of suitable sensitizers. Moreover, the Laporte‐forbidden nature of these transitions, together with energy‐level mismatch, results in intrinsically low excitation efficiencies. While broadband excitation spanning the ultraviolet to NIR would provide unprecedented flexibility in excitation wavelength selection and unlock substantial application potential, as demonstrated in information security using multi‐dye‐sensitized upconverting nanoparticles [41], achieving full‐spectrum excitation for NIR‐II‐emissive DSNPs remains a formidable challenge.

**FIGURE 1:**
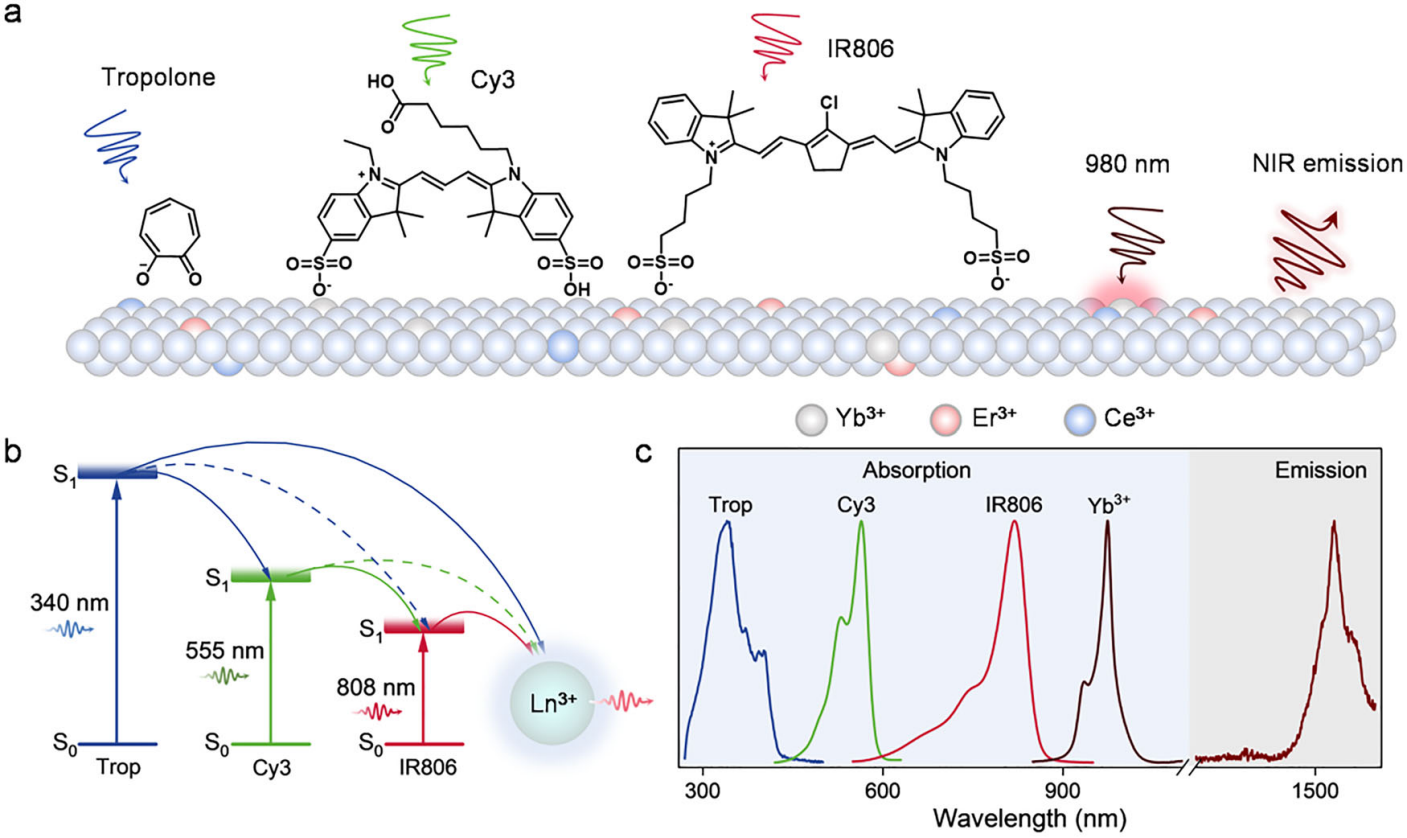
Multiple‐dye‐sensitized DSNPs. (a) Molecular structures of the three dyes (tropolone, Cyanine 3, and IR806). (b) Schematic illustration of their coordination to DSNPs, showing the cascade energy‐transfer pathway. (c) Absorption spectra of the three dyes across the ultraviolet–visible–near‐infrared (UV–Vis–NIR) range (270–1030 nm) and the NIR‐II emission spectrum of DSNPs, demonstrating broadband excitability.

Here, we introduce a cascade sensitization strategy that enables multi‐band‐driven NIR‐II emission from DSNPs through the integration of multiple organic dyes. Surface modification of NaYF_4_:Yb^3+^, Ce^3+^, Er^3+^ DSNPs with tropolone (Trop), Cyanine 3 (Cy3), and IR806 collectively broadens the absorption window from the ultraviolet to the near‐infrared region (270–1030 nm) by sixfold, resulting in intense NIR‐II emission at 1525 nm (Figure 1). While Trop and IR806 individually exhibit efficient energy transfer to DSNPs (63% and 96%, respectively), Cy3 alone showed limited sensitization efficiency (∼ 6%). Notably, IR806 further acts as an energy‐transfer bridge, enabling cascaded energy‐transfer processes that markedly enhance NIR‐II emission for Cy3 excitation (from ∼6% to 70% efficiency). Furthermore, this modular dye‐sensitization strategy enables tunable NIR‐II emission over a broad spectral range from 1060 nm to 1525 nm. By overcoming the traditional excitation limitations of DSNPs, the resulting broadband excitability and emission tunability provide unprecedented flexibility in excitation and detection wavelength selection, opening new opportunities for advanced bioimaging, multiplexed sensing, and photonic applications.

## 2 Results and Discussion

**FIGURE 2:**
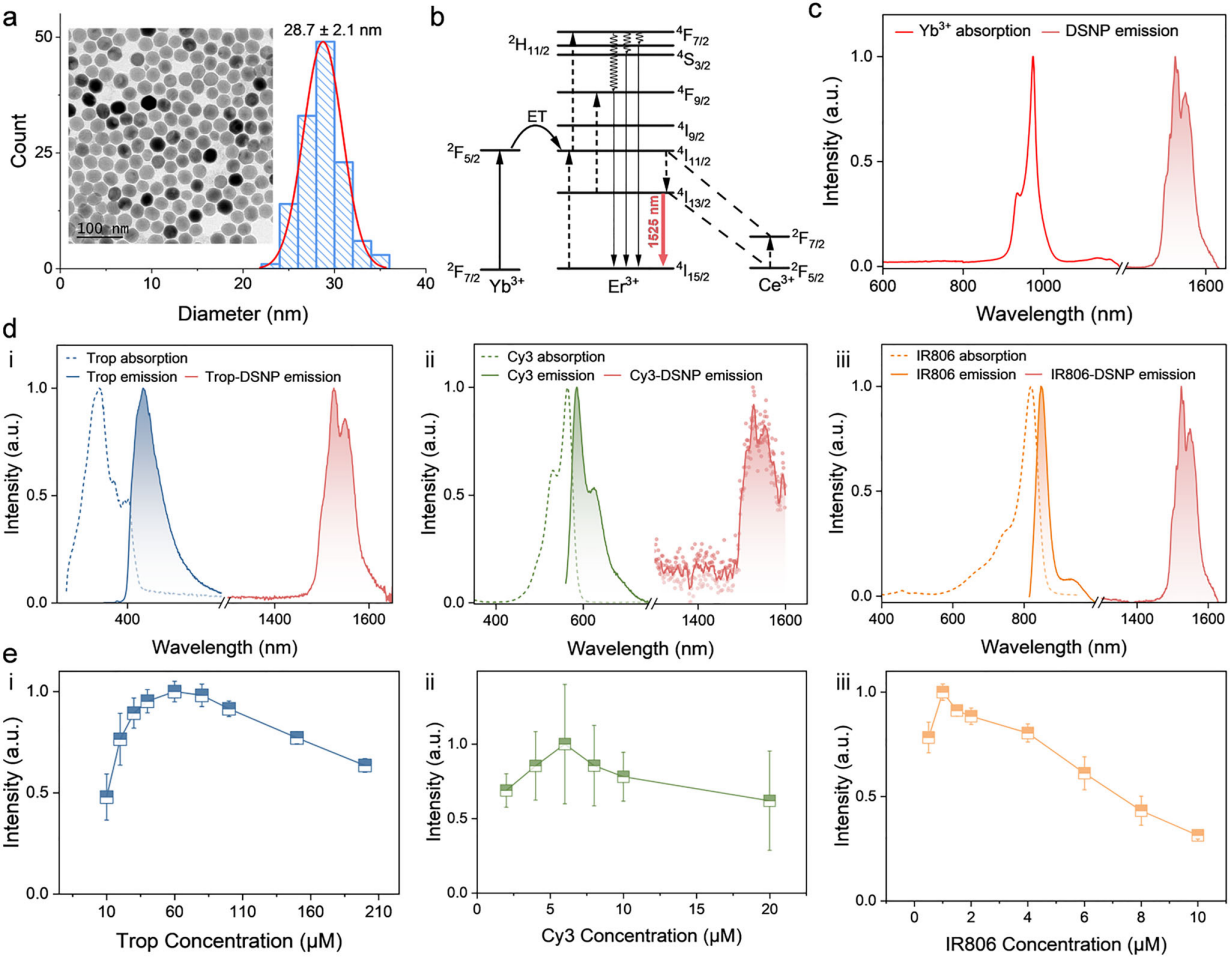
Structural and optical characterization of DSNPs and dye‐DSNPs systems. (a) TEM image of DSNPs and corresponding size distribution, showing an average diameter of 28.7 ± 2.1 nm. (b) Energy‐level diagram illustrating energy transfer between Yb^3+^, Er^3+^, and Ce^3+^ ions. (c) Normalized emission spectrum of DSNPs and absorption spectrum of Yb^3+^ ions. (d) Absorption and emission spectra of three organic dyes, alongside the NIR‐II emission spectra of dye‐sensitized DSNPs under excitation at 340, 555, and 808 nm, respectively: (i) Trop; (ii) Cy3; (iii) IR806. (e) Dependence of NIR‐II emission intensity of the dye‐DSNPs systems on dye concentration: (i) Trop; (ii) Cy3; (iii) IR806.

A high‐temperature solvothermal method was employed to synthesize the NaYF_4_: 20% Yb^3+^, 2% Er^3+^, 10% Ce^3+^ DSNPs, yielding uniform spherical nanoparticles with a narrow size distribution and an average diameter of ∼29 nm (Figure 2a). Prior to dye functionalization, the oleic acid ligands were removed to expose the particle surface. Ce^3+^ doping was introduced to enhance downshifting via promotion of cross‐relaxation pathways.

Figure 2b illustrates the energy transfer processes among Yb^3+^, Er^3+^, and Ce^3+^ ions. Under 980 nm excitation, Yb^3+^ ions act as sensitizers, absorbing photons and populating the ^2^F_5/2_ state, which transfers energy to the ^4^I_11/2_ level of Er^3+^, via phonon‐assisted processes. This compensates for the small energy mismatch between the ^2^F_5/2_ state of Yb^3+^ and the ^4^I_11/2_ state of Er^3+^ through coupling between dopant ions and lattice phonons [42]. The long‐lived ^4^I_11/2_ state allows further excitation of Er^3+^ ions to the higher‐lying ^4^F_9/2_ and ^4^F_7/2_ states. Subsequent non‐radiative relaxation populates the ^2^H_11/2_, ^4^S_3/2_, and ^4^F_9/2_ levels, which give rise to the up‐conversion emission at 525 nm, 541 nm, and 665 nm, via radiative transitions to the ground ^4^I_15/2_ state. In parallel, excitons in the ^4^I_11/2_ state can undergo non‐radiative relaxation to the ^4^I_13/2_ level, followed by NIR‐II emission at 1525 nm (^4^I_13/2_ → ^4^I_15/2_). Visible up‐conversion and NIR‐II downshifting emissions are competing processes. The energy difference between ^4^I_11/2_ and ^4^I_13/2_ of Er^3+^ (3700 cm^−1^) closely matches the energy gap between ^2^F_5/2_ and ^2^F_7/2_ levels of Ce^3+^ (2300 cm^−1^), enabling efficient cross‐relaxation mediated by Ce^3+^. This pathway accelerates depopulation of the ^4^I_11/2_ state and increases the population of the ^4^I_13/2_, making the NIR‐II at 1525 nm dominant while suppressing visible up‐conversion (Figures 2c and S1).

A two‐step ligand exchange strategy was used to graft organic dyes onto the DSNP surface. Three dyes—tropolone (Trop), Cyanine (Cy3), and IR806—were chosen based on their complementary absorption range and negative charge for coordination to the positively charged DSNP. Each dye contains an aromatic chromophore for light harvesting and a carboxylic acid moiety for robust coordination (Figure 1a). Figure 2d compares the absorption and emission spectra of the dyes. Trop exhibited a broad absorption in the ultraviolet‐visible region (270–430 nm), with a peak at 340 nm (full width at half maximum (FWHM): ∼72 nm, absorption coefficient: 14962 M^−1^ cm^−1^). Cy3 absorbs predominantly in the visible (420–600 nm) with a peak at 565 nm (FWHM ∼56 nm, 129710 M^−1^ cm^−1^). IR806 absorbs strongly in near‐infrared (600–900 nm), peaking at ∼800 nm (218377 M^−1^ cm^−1^) with FWHM of ∼55 nm.

Although Trop showed no direct spectral overlap with the absorption bands of Yb^3+^, intense NIR‐II emission was observed under 340 nm excitation, indicating efficient energy transfer from Trop to the DSNPs. The presence of DSNPs led to a markedly quenched fluorescence of Trop, corresponding to an energy‐transfer efficiency of approximately 63% (Figure S2a). This nonconventional sensitization efficiency is likely associated with the triplet‐mediated energy transfer pathway. The triplet state of Trop (16800 cm^−1^) has been reported to be compatible for efficient energy transfer with the low‐lying accepting levels of several Ln^3+^ ions, such as Nd^3+^, Er^3+^, Ho^3+^ and Tm^3+^ [43]. Although the energy gap between the Trop triplet state (16800 cm^−1^) and ^2^F_5/2_ level of Yb^3+^ (10200 cm^−1^) is relatively large (∼6600 cm^−1^), energy transfer to Yb^3+^ can still proceed efficiently via phonon‐assisted relaxation [44, 45, 46]. In contrast, Cy3 has been reported to exhibit a low triplet quantum yield [47], which limits its ability to sensitize Yb^3+^. Cy3 sensitization resulted in only weak NIR‐II emission, accompanied by negligible quenching of Cy3 fluorescence upon conjugation with DSNPs (Figure S2b), with an inefficient energy‐transfer efficiency of ∼6%.

In sharp contrast, IR806 exhibited highly efficient sensitization of DSNPs, with an energy‐transfer efficiency of 96% (Figure S2c). Notably, energy transfer from IR806 to DSNPs can proceed through both singlet‐ and triplet‐mediated pathways [48]. The effect of dye loading on the NIR‐II emission was systematically investigated. Trop‐sensitized DSNPs showed increasing NIR‐II emission intensity with concentration, reaching a maximum at 60 µM (Figures 2e‐i and S3a). In contrast, Cy3 exhibited weak dependence due to low intrinsic sensitization efficiency, with an optimal Cy3 concentration of 6 µM (Figures 2e‐ii and S3b). IR806‐DSNPs displayed a peak emission at 1 µM, followed by concentration quenching at higher loadings (Figures 2e‐iii and S3c).

Following the systematic evaluation of individual sensitizers (Trop, Cy3, and IR806), we next investigated their interactions and effects on NIR‐II emission in the dual‐dye‐sensitized DSNPs systems. Because all sensitizers are confined to the limited surface area of the DSNPs, an excessive amount of dye may lead to competitive surface loading on the nanoparticles. To mitigate this effect, we changed the conjugation protocol by reducing the loading of each dye to half of its optimal concentration determined for the corresponding single‐dye‐sensitized DSNP systems, as this condition yielded a higher emission intensity than the original concentration (Figure S4). The dye‐to‐DSNP ratios were estimated to be 938:1, 94:1, and 16:1 for Trop, Cy3, and IR806, respectively (Supporting Information).

**FIGURE 3:**
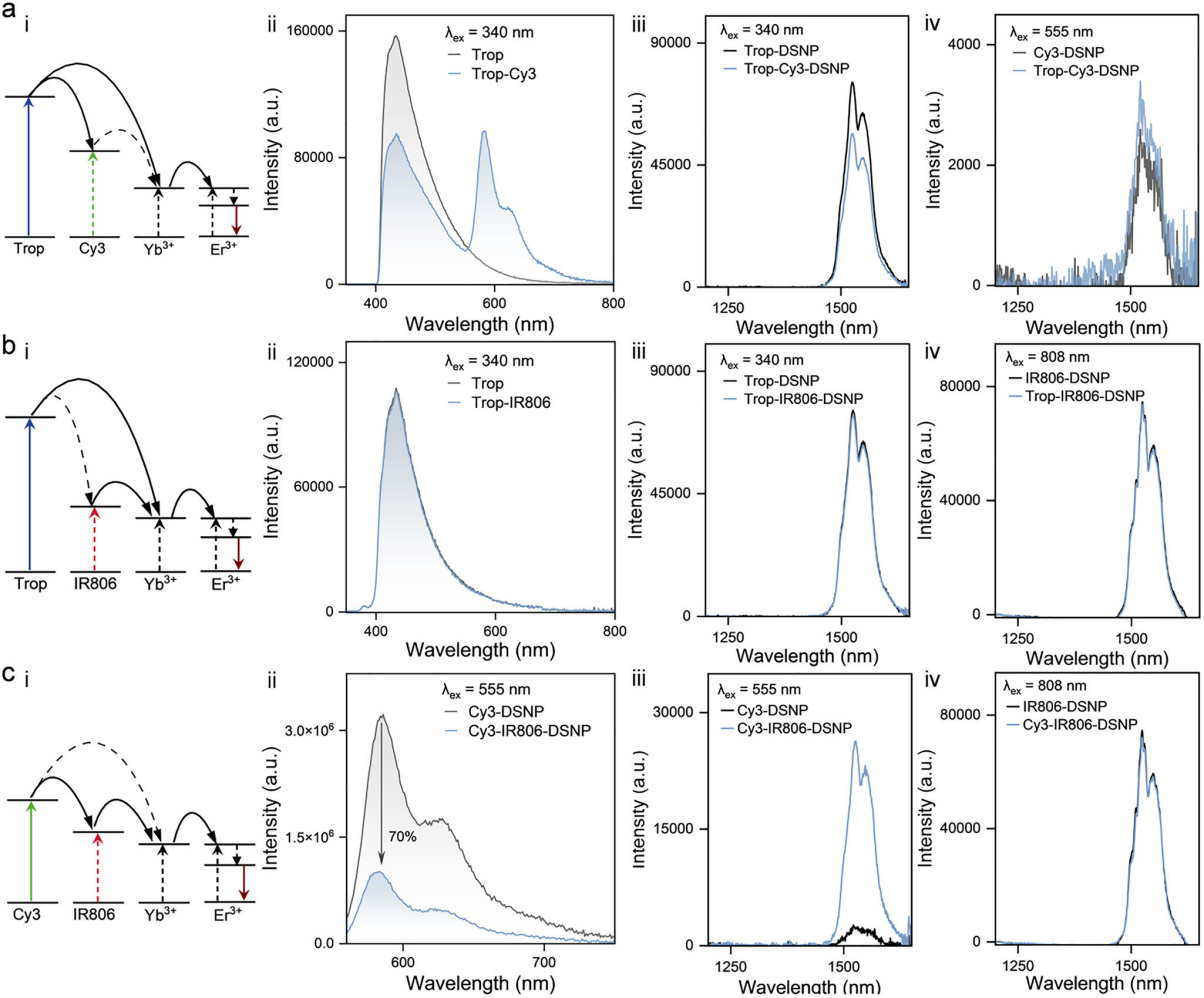
Dual‐dye‐sensitized DSNP systems. (a) Trop‐Cy3‐DSNPs: (i) Schematic illustration of Trop‐Cy3‐DSNPs system; (ii) Emission spectra of Trop and Trop‐Cy3 systems (*λ*_ex_ = 340 nm); (iii) Comparison of NIR‐II emission intensities of Trop‐DSNPs and Trop‐Cy3‐DSNPs systems under 340 nm excitation; (iv) Comparison of NIR‐II emission intensities of Cy3‐DSNPs and Trop‐Cy3‐DSNPs under 555 nm excitation. (b) Trop‐IR806‐DSNPs: (i) Schematic illustration of Trop‐IR806‐DSNPs system. (ii) Emission spectra of Trop and Trop‐IR806 systems (*λ*_ex_ = 340 nm); (iii) Comparison of NIR‐II emission intensities of Trop‐DSNPs and Trop‐IR806‐DSNPs under 340 nm excitation; (iv) Comparison of NIR‐II emission intensities of IR806‐DSNPs and Trop‐IR806‐DSNPs under 808 nm excitation; (c) Cy3‐IR806‐DSNPs: (i) Schematic illustration of Cy3‐IR806‐DSNPs system; (ii) Emission spectra of Cy3 in the Cy3‐DSNPs and Cy3‐IR806‐DSNPs systems (*λ*_ex_ = 555 nm); (iii) Comparison of NIR‐II emission intensities of Cy3‐DSNPs and Cy3‐IR806‐DSNPs systems under 555 nm excitation; (iv) Comparison of NIR‐II emission intensities of IR806‐DSNPs and Cy3‐IR806‐DSNPs under 808 nm excitation.

Figures 3a‐i illustrated the Trop‐Cy3 dual‐dye‐sensitized DSNP system. Notably, substantial spectral overlap exists between Trop emission and Cy3 absorption (Figure S5), providing a favorable condition for energy transfer from Trop to Cy3. Upon 340 nm excitation, which selectively excites Trop, the Trop‐Cy3 system displayed the characteristic emission features of both dyes (Figure 3a‐ii). The observation of Cy3 emission was mainly attributed to the absorption band of Cy3 at around 340 nm (Figure S6) and its direct excitation at this wavelength (Figure S7). In contrast, under 400 nm excitation—where the Cy3 absorption is minimal—the Cy3 emission was significantly enhanced by 2.4‐fold compared to Cy3 alone (Figure S8), confirming the effective energy transfer from Trop to Cy3. Despite this efficient inter‐dye energy transfer, introduction of Cy3 into Trop‐sensitized DSNPs resulted in a moderate decrease (∼25%) in the NIR‐II emission intensity at 1525 nm (Figure 3a‐iii). This attenuation is attributed to partially transferred excitation energy to Cy3, followed by inefficient subsequent energy transfer from Cy3 to the DSNPs, consistent with the relatively low sensitization efficiency of Cy3 observed in single‐dye systems (Figure 2d). Furthermore, under direct excitation at 555 nm, the NIR‐II emission intensity of Cy3‐DSNPs remained weak and essentially unchanged upon incorporation of Trop (Figures 3a‐iv and S9), indicating negligible back energy transfer from Cy3 to the higher‐energy Trop chromophore.

In contrast, the Trop‐IR806 dual‐dye system exhibited fundamentally different energy transfer behavior. The singlet excited‐state of Trop (3.65 eV) is substantially higher than that of the IR806 (1.53 eV) (Figure 3b‐i) and negligible spectral overlap was observed between Trop emission and IR806 absorption (Figure S10), precluding efficient energy transfer between the two dyes. This conclusion is corroborated by their fluorescence measurements. Under 340 nm excitation, only the characteristic emission of Trop (∼450 nm) was detected, with no observable contribution from IR806 (Figure 3b‐ii). Further evidence was provided by the NIR‐II emission behavior of the Trop‐IR806‐DSNPs. Consistent with these observations, excitation at either 340 nm (Trop selective) or 808 nm (IR806 selective) yielded NIR‐II emission at 1525 nm that was comparable to that of the corresponding single‐dye‐sensitized DSNP systems (Figures 3b‐iii, 3b‐iv, S11a, and S11b). This invariance confirms that the Trop and IR806 independently sensitized the DSNPs, with no detectable inter‐dye energy transfer.

The Cy3‐IR806 dual‐dye system was next investigated (Figure 3c). Although Cy3 alone exhibited negligible sensitization efficiency toward DSNPs, a significant 10‐fold enhancement was observed in the Cy3‐IR806‐DSNPs system upon excitation of Cy3 (Figures 3c‐iii). This behavior originates from an efficient cascade energy transfer mechanism. The singlet excited‐state energies of Cy3 and IR806 are 2.23 and 1.53 eV, respectively, and substantial spectral overlap exists between the emission of Cy3 and the absorption of IR806 (Figure S12). Consequently, excitation energy absorbed by Cy3 can be efficiently transferred to adjacent IR806 molecules. The excited‐state energy of IR806 is well matched with absorption of Yb^3+^ ions and thereby enhancing the NIR‐II emission intensity of DSNPs (Figure 3c‐i). Whereas conjugation of Cy3 with DSNPs alone induced negligible fluorescence quenching, incorporation of IR806 resulted in pronounced quenching of Cy3 emission, corresponding to an energy‐transfer efficiency of approximately 70% (Figure 3c‐ii). The fluorescence quenching of Cy3 and the emerging of IR806 emission in the Cy3‐IR806 dual dye system further confirm the presence of efficient inter‐dye energy transfer (Figure S13a). The fluorescence lifetime of Cy3 in Cy3‐DSNPs decreased from 1.11 ns to 0.45 ns upon the integration of IR806, indicating the occurrence of nonradiative energy transfer between the two molecules (Figure S13b). Under 808 nm excitation, the NIR‐II emission intensities of the IR806‐DSNPs and Cy3‐IR806‐DSNPs are nearly identical, suggesting negligible back energy transfer from IR806 to Cy3 (Figures 3c‐iv and S11b).

**FIGURE 4:**
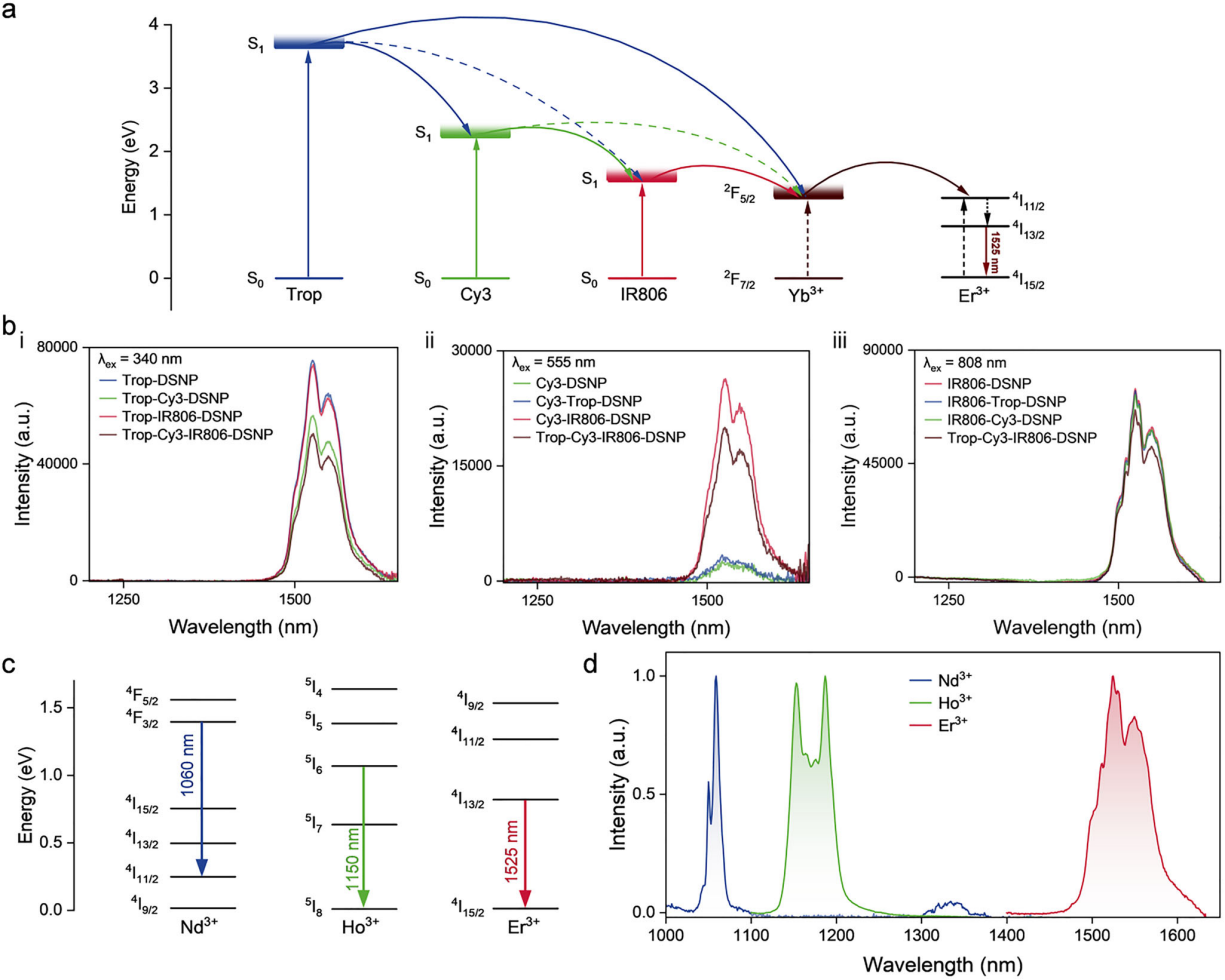
Triple‐dye‐sensitized DSNPs systems and tunable NIR‐II emission. (a) Schematic illustration of the cascaded energy transfer process in the Trop‐Cy3‐IR806‐DSNPs system. (b) Comparison of NIR‐II emission intensities of single‐dye‐sensitized DSNPs, dual‐dye‐sensitized DSNPs, and triple‐dye‐sensitized DSNPs under excitation at (i) 340 nm, (ii) 555 nm, (iii) 808 nm. (c) Energy‐level diagrams of Nd^3+^, Ho^3+^, and Er^3+^ illustrating their characteristic NIR‐II emission transitions. (d) Representative NIR‐II emission spectra of Nd^3+^‐, Ho^3+^‐, and Er^3+^‐doped DSNPs, demonstrating tunable emission under triple‐dye sensitization.

Following the investigation of dual‐dye‐sensitized DSNP systems, we extended the study to triple‐dye sensitization. The Trop‐Cy3‐IR806‐DSNPs were prepared via sequential ligand‐exchange reactions (Figure 4a), and their NIR‐II emission intensities were systematically compared with those of single‐ and dual‐dye‐sensitized counterparts (Figure 4b). Consistent with the negligible energy transfer from Trop to IR806, the NIR‐II emission of Trop‐IR806‐DSNPs remained essentially unchanged relative to the corresponding single‐dye systems. In contrast, both Trop‐Cy3‐DSNPs and Trop‐Cy3‐IR806‐DSNPs exhibited a slight decrease in NIR‐II emission, which can be attributed to partial energy transfer from the highly efficient Trop sensitizer to the less efficient Cy3 (Figure 4b‐i).

Upon excitation at 555 nm, corresponding to the absorption maximum of Cy3, both Cy3‐DSNPs and Trop‐Cy3‐DSNPs displayed the weakest NIR‐II emission, reflecting the intrinsically low energy‐transfer efficiency from Cy3 to DSNPs and the absence of back energy transfer from Cy3 to Trop. In contrast, incorporation of IR806 led to a pronounced enhancement in NIR‐II emission in both Cy3‐IR806‐DSNPs and Trop‐Cy3‐IR806‐DSNPs, underscoring the critical role of IR806 as an energy‐transfer bridge in the cascade sensitization pathway (Figure 4b‐ii). Under 808 nm excitation, which was chosen to excite IR806, the NIR‐II emission intensities of IR806‐sensitized DSNPs showed minimal variation regardless of whether single, dual, or triple dyes are present (Figure 4b‐iii), confirming the absence of back energy transfer from IR806 to Cy3 or Trop.

This multiple dye sensitization strategy markedly broadened the excitation window of DSNPs across the ultraviolet–visible–near‐infrared (UV–Vis–NIR) region, spanning 270–1030 nm, achieving approximately 5.8‐fold and 13.8‐fold increases compared with the intrinsic Yb^3+^ (980 nm) and Nd^3+^ (808 nm) excitation bands (Figure S14). The energy transfer pathways in Trop‐Cy3‐IR806‐DSNPs system are illustrated in Figure 4a. Efficient spectral overlap between Trop and Cy3, as well as between Cy3 and IR806, enables sequential energy relay through the dye cascade, and ultimately transferring excitation energy to Yb^3+^ (Figures S5 and S12). Notably, incorporation of IR806 dramatically enhanced the NIR‐II emission under 555 nm excitation, where Cy3 alone is an inefficient sensitizer. These results demonstrate that cascaded energy transfer combined with synergistic dye sensitization underpins the expanded excitation window.

Lanthanide activators provide a rich palette of NIR‐II emission wavelengths, forming a strong foundation for multiplexed optical applications. By selecting different lanthanide ions, the emission wavelength of DSNPs can be tuned under the same multi‐band excitation conditions enabled by the triple‐dye sensitization strategy (Figure 4c,d). To demonstrate this tunability, we synthesized Nd^3+^‐doped DSNPs (Figure S15) and Ho^3+^‐doped DSNPs (Figure S16), which exhibited characteristic NIR‐II emissions at 1060 nm (^4^F_3/2_ → ^4^I_11/2_) and 1150 nm (^5^I_6_ → ^5^I_8_), respectively. Both systems retained broadband excitability across the ultraviolet, visible, and near‐infrared regions (Figures S17 and S18), validating the universality and robustness of the multiple‐dye sensitization approach. Beyond extending the excitation window, this strategy allows modular tuning of NIR‐II emission through rational lanthanide doping (Figure 4c,d), providing access to diverse emission channels without altering the excitation source. Consequently, the emission wavelength can be readily selected and expanded according to specific application requirements, such as spectral multiplexing, imaging [28], or multichannel sensing. Collectively, these results establish multiple‐dye‐sensitized DSNPs as a versatile platform for broadband‐excitable, wavelength‐programmable NIR‐II nanoprobes. Benefiting from the modular design of the system, the nanoparticle platform can be readily adapted for both deep‐tissue imaging under NIR excitation and imaging in low‐penetration‐depth regions under UV and visible excitation, where light penetration depth is not a limiting factor [49, 50].

## 3 Conclusion

In summary, we established a cascade multiple‐dye sensitization strategy that enables lanthanide‐doped DSNPs to be efficiently excited across the entire UV–Vis–NIR window (270–1030 nm) while retaining strong and tunable NIR‐II emission. By rationally matching energy levels and spectral overlap among Trop, Cy3, and IR806, excitation energy was relayed unidirectionally toward the lanthanide lattice, with IR806 acting as a critical bridge that converts otherwise inefficient visible excitation into intense NIR‐II output. Beyond broadband excitation, this strategy enables modular tuning of NIR‐II emission wavelengths through rational selection of lanthanide activators, as demonstrated for Er^3+^, Nd^3+^, and Ho^3+^ systems, without altering the excitation source. The universality and robustness of this approach position multiple‐dye‐sensitized DSNPs as a versatile platform for wavelength‐programmable NIR‐II photonics, opening new opportunities in multiplexed imaging, optical sensing, and advanced photonic applications.

## Author Contributions

**Lijun Jiang**: writing – review and editing. **Yuxia Luo**: writing – original draft, funding acquisition. **Yunge Du**: data curation, writing – original draft, investigation. **Chengyi Chu**: resources. **Guochen Bao**: conceptualization, writing – review and editing, supervision, funding acquisition. **Yin Huang**: writing – review and editing.

## Conflicts of Interest

The authors declare no conflicts of interest.

## Supporting information




**Supporting File 1**: anie72650‐sup‐0001‐SuppMat.pdf.

## Data Availability

The data that support the findings of this study are available from the corresponding author upon reasonable request.
