# Supplementary material for: Multi‐Band Excitation of NIR‐II Lanthanide Nanoparticles via Multiple‐Dye Cascade Sensitization
Source: Angew Chem Int Ed Engl. 2026 May 17;65(26):e2349085. doi: 10.1002/anie.2349085 (PMC13285467; doi:10.1002/anie.2349085)
Supplement: Supplementary file 1 — Supporting File 1: anie72650‐sup‐0001‐SuppMat.pdf. [file ANIE-65-e2349085-s001.pdf]

Supporting information for

Multi-Band Excitation of NIR-II Lanthanide Nanoparticles via Multiple-Dye Cascade Sensitization

Yuxia Luo[1*,#], Yunge Du[1,#], Yin Huang[2], Lijun Jiang[3], Chengyi Chu[4], Guochen Bao[2,5*]

[1] College of Bioresources Chemical and Materials Engineering, Shaanxi University of Science & Technology, Xi'an 710021, Shaanxi, China
[2] School of Mathematical and Physical Sciences, Faculty of Science, University of Technology Sydney, Sydney, New South Wales 2007, Australia
[3] Key Laboratory of Pesticide & Chemical Biology of Ministry of Education, Hubei Key Laboratory of Genetic Regulation and Integrative Biology, School of Life Sciences, Central China Normal University, Wuhan 430079, China
[4] Laboratory of Atomic-scale and Micro & Nano Manufacturing, Ningbo Institute of Materials Technology and Engineering, Chinese Academy of Sciences, Ningbo 315201, China
[5] Institute for Biomedical Materials and Devices (IBMD), Faculty of Science, University of Technology Sydney, Sydney, New South Wales 2007, Australia
Email: guochen.bao@uts.edu.au; E-mail: luoyuxia@sust.edu.cn
[#] These authors contributed equally to the work

A. Experiment procedures

1. Reagents

Hydrated rare earth chlorides ($YCl_3·6H_2O$ 99.9%, $YbCl_3·6H_2O$ 99.9%, $ErCl_3·6H_2O$ 99.9%, $NdCl_3·6H_2O$ 99.9%, $HoCl_3·6H_2O$ 99.9%, $CeCl_3·7H_2O$ 99.9%), Tropolone, Sodium hydroxide (NaOH, ⩾98%), Ammonium fluoride (NH_4F, 96%), Cyclohexane (99.5%), Oleic acid (OA, 90%), 1-Octadecene (ODE, 90%), Methanol (⩾99.9%) were purchased from Shanghai Aladdin Biochemical Technology Co., Ltd. IR806 was purchased from Sigma Aldrich. Toluene was purchased from Sinopharm Chemical Reagent Co., Ltd. Cyanine 3 (Cy3), N,N-Dimethylformamide (DMF), Nitrosyl tetrafluoroborate ($NOBF_4$), and Absolute ethanol (95%) were purchased from Shanghai Macklin Biochemical Technology Co., Ltd. All chemicals were used as received without any further purification.

2. Synthesis of DSNPs

DSNPs ($NaYF_4$: 20% Yb^{3+}, 2% Er^{3+}, 10% Ce^{3+}) were synthesized using a high-temperature solvent method, with specific methods according to literature[1]. The synthesis process was as follows: Yttrium Chloride (YCl_3, 0.68 mmol), Ytterbium Chloride ($YbCl_3$, 0.2 mmol), Erbium Chloride ($ErCl_3$, 0.02 mmol), and Cerium Chloride ($CeCl_3$, 0.1 mmol) were dissolved in a mixed solvent of 6 mL of Oleic acid (OA) and 15 mL of 1-octadecene (ODE). The mixture solution was heated to 150 °C for 30 min under vacuum to remove methanol and water to form a homogeneous rare-earth-oleate precursor. Subsequently, the solution was cooled to room temperature. A mixture solution of ammonium fluoride (NH_4F, 4 mmol) and sodium hydroxide (NaOH, 2.5 mmol) in 10 mL of methanol was added, and the mixture was stirred at room temperature for 30 min. Then, the mixture was heated to 150 °C under vacuum and maintained at this temperature for another 30 min to thoroughly remove residual water and methanol. After the bubbles disappeared completely, the solution was heated to 300 °C under an argon atmosphere and kept for 90 min, then naturally cooled down to room temperature. The DSNPs were precipitated by adding ethanol, and then centrifuged at 10,000 rpm for 15 min. The DSNPs were redispersed in cyclohexane, precipitated by ethanol, and then centrifuged at 10,000 rpm for 15

min. The process was performed three times. Finally, the DSNPs were dispersed in cyclohexane for further characterization.

$NaYF_4$: 20% Nd^{3+}: Yttrium Chloride (YCl_3, 0.8 mmol) and Neodymium Chloride ($NdCl_3$, 0.2 mmol) were dissolved in a mixed solvent consisting of 6 mL of OA and 15 mL of ODE. The subsequent reaction, and purification steps were performed as described above.

$NaYF_4$: 40% Yb^{3+}, 2% Ho^{3+}: Yttrium Chloride (YCl_3, 0.58 mmol), Ytterbium Chloride ($YbCl_3$, 0.4 mmol), and Holmium Chloride ($HoCl_3$, 0.02 mmol) were dissolved in a mixed solvent of 6 mL of OA and 15 mL of ODE. The following reaction, separation, and purification procedures were carried out in the same manner as described above.

3. Preparation of Dye-Sensitized DSNPs

OA was removed from the surface of DSNPs by nitrosonium tetrafluoroborate ($NOBF_4$)[2]. OA-coated DSNPs dispersed in cyclohexane were mixed with a DMF solution of $NOBF_4$ (1 mg/mL). The mixture was shaken for 15 minutes and left to stand for 20 minutes to extract the DSNPs into the DMF layer. The supernatant was then removed, and cyclohexane and toluene (1:1) were added to the DMF layer, followed by centrifugation at 10,000 rpm for 15 min. The precipitated DSNPs were redispersed in DMF and then diluted with DMF to 1 mg/mL for dye sensitization experiments.

500 μmol/L dye solutions (Trop, Cy3 and IR806) in DMF were prepared for further dye-sensitization experiments. Different amounts of organic fluorescent dye solution were added to the DSNPs and vortexed for 5 min. For the triple-dye-sensitized DSNP system, the three dyes were co-loaded simultaneously under identical conditions following OA removal.

4. Characterization and measurement

Absorption spectra: The absorption spectra were measured using an Agilent Cary 5000 UV-Vis-NIR spectrophotometer. The procedure was as follows: first, the sample was dissolved in the corresponding solvent and transferred to a quartz cuvette, which was then placed in the

liquid sample compartment. Before the measurement, a blank spectrum of the pure solvent was collected under the same conditions as the background. The sample was then scanned to obtain its spectrum. Finally, the absorption spectrum of the sample was obtained after automatic baseline correction.

Solutions of Trop, Cy3, and IR806 with concentrations of 5 μmol/L were prepared using DMF as the solvent, and their absorption spectra were measured. Similarly, solutions of Yb^{3+} and Nd^{3+} with concentrations of 0.2 mmol/L were prepared using methanol as the solvent, and their absorption spectra were also measured.

Emission spectrum and lifetime measurement: Emission spectra were measured using an FLS 1000 fluorescence spectrometer (Edinburgh Instruments, UK). It was equipped with a Near-Infrared Photomultiplier Tube (NIR-PMT), which extends spectral coverage up to 1700 nm. For different sample systems, corresponding excitation protocols were applied: for bare downshifting nanoparticles, a 980 nm continuous-wave laser (7 W/cm^2) was used as the excitation source to record their upconversion and downshifting luminescence signals within specific wavelength ranges; for dye-sensitized downshifting nanoparticles, a 450 W xenon lamp equipped in the spectrometer or an 808 nm continuous-wave laser (3.7 W/cm^2) was used as the excitation source to obtain emission spectra. The power density of excitation sources was measured using a VLP-2000 optical power meter. The luminescence lifetime was measured on the spectrometer. A pulsed diode laser (EPL-510, λ_{ex} = 510 nm) was employed as the excitation source, and the fluorescence intensity decay was recorded using the time-correlated single photon counting (TCSPC) technique to obtain the time-resolved fluorescence decay curves.

Transmission electron microscope (TEM): The morphology, average size, and particle size distribution of the synthesized DSNPs were characterized using Tecnai F20 and Hitachi HT7820 TEM with an acceleration voltage of 100 kV. The sample preparation process was as follows: first, DSNPs were dispersed in cyclohexane, and an appropriate amount of the dispersion was diluted with cyclohexane. Ultrasonic treatment was performed for 1 hour to obtain a

homogeneous suspension. Then, 10 μL of this suspension was dropped onto an ultrathin carbon-coated copper grid, which was dried overnight at room temperature to allow sufficient solvent evaporation. Before TEM imaging, the copper grid was further dried under an infrared lamp to completely remove residual solvent.

5. Data Processing and Calculation

Molecular weight. The average diameter of DSNPs is 28.7 nm. The nanoparticle volume is estimated to be as 12379 nm^3 based on a spherical approximation. The density of $NaYF_4$ has been reported[3] as 4.21 g/cm^3 and we use this value to estimate the average mass of a single DSNP which is calculated to be 12,379 $nm^3 \times (4.21\times10^{-21}$ $g/nm^3) = 5.21\times10^{-17}$ g. Accordingly, the average molecular weight (MW_{DSNP}) is estimated to be 3.14×10^{7} g/mol by multiplying by Avogadro's constant (6.02×10^{23}).

Dye-to-nanoparticle ratio. The DSNP concentration employed in this study is 1 mg/mL, corresponding to ca. 0.032 μM (calculated as (1 g/L)/(3.14×10^{7} g/mol)). Based on dye concentrations of 30 μM, 3 μM, and 0.5 μM, the average numbers of dye molecules per particle are estimated to be 938, 94, and 16, respectively.

Energy transfer efficiency (ETE). The ETE was determined by comparing the integrated emission intensities of the dyes in the absence and presence of acceptor. The integration ranges for the characteristic emission peaks were set as 350–600 nm for Trop, 560–750 nm for Cy3, and 815–1000 nm for IR806. The ETE was calculated using the following equation:

$$ETE(\%)=\frac{I_D\text{-}I_{DA}}{I_D}\times 100\%$$

where I_D represents the integrated emission area of the dye alone, and I_{DA} represents the integrated emission area upon the addition of acceptor. All measurements were performed under identical instrumental settings and experimental conditions to ensure comparability.

B. Supporting figures

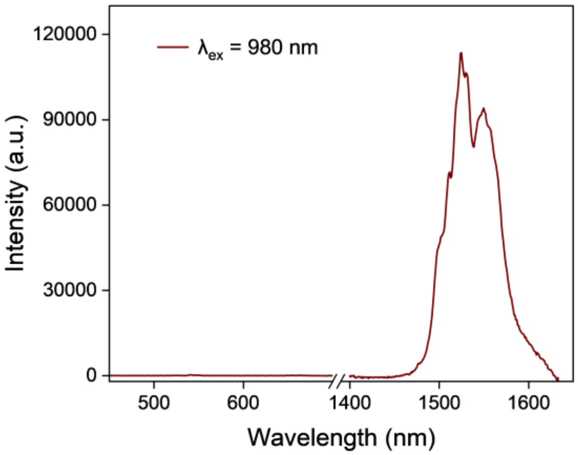

Figure S1. Upconversion and downshifting emission spectra of $NaYF_4$: 20% Yb^{3+}, 2% Er^{3+}, 10% Ce^{3+} under the excitation of 980 nm laser. DSNPs concentration: 1 mg/mL.

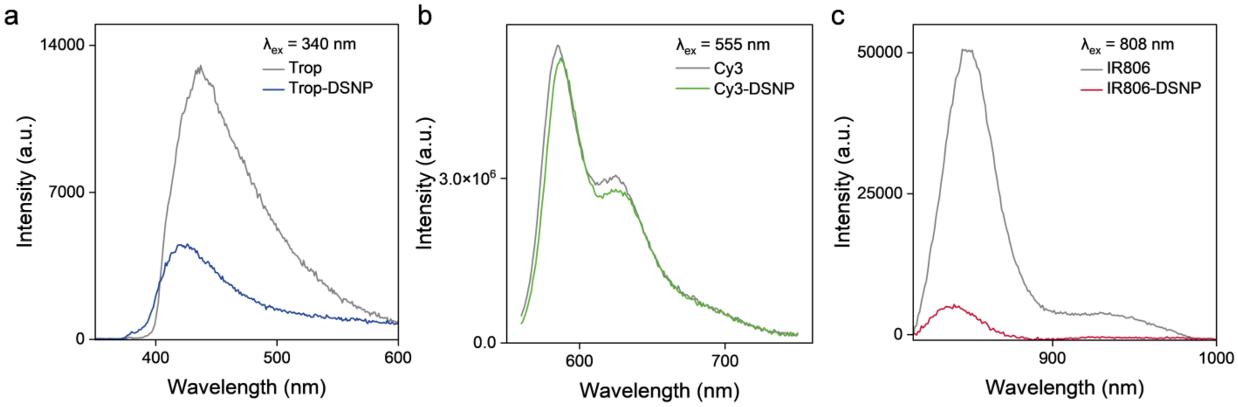

Figure S2. Emission spectra of dyes before and after the incorporation with DSNPs under the excitation of 340, 555, and 808 nm for Trop, Cy3, and IR806.

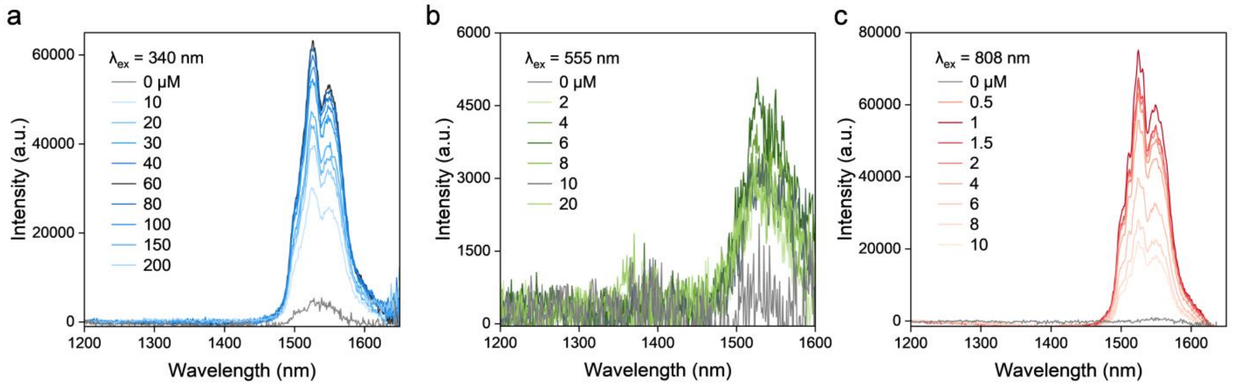

Figure S3. NIR-II emission intensity of the dye-DSNPs systems as a function of dye concentration under the excitation of 340, 555, and 808 nm (a. Trop; b. Cy3; c. IR806).

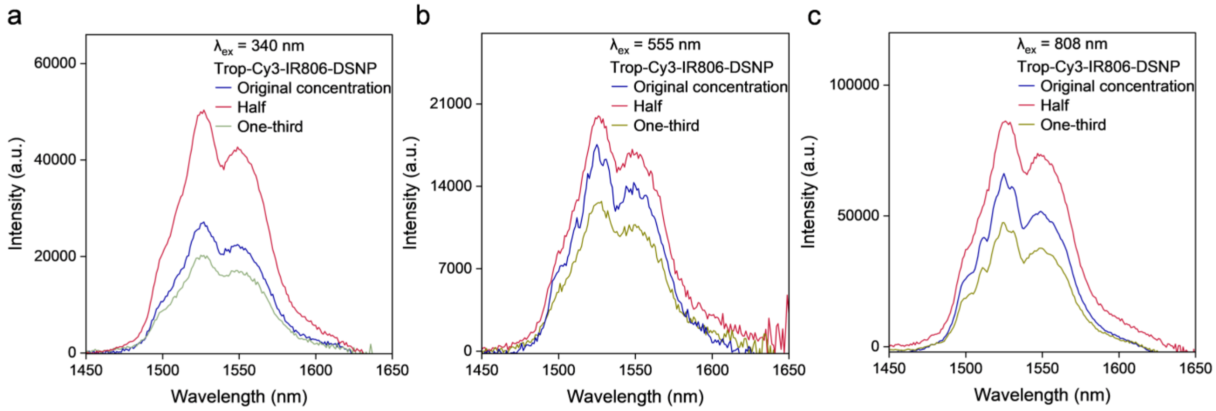

Figure S4. The emission spectra of Trop-Cy3-IR806-DSNPs with different concentrations excited at (a) 340 nm, (b) 555 nm, and (c) 808 nm.

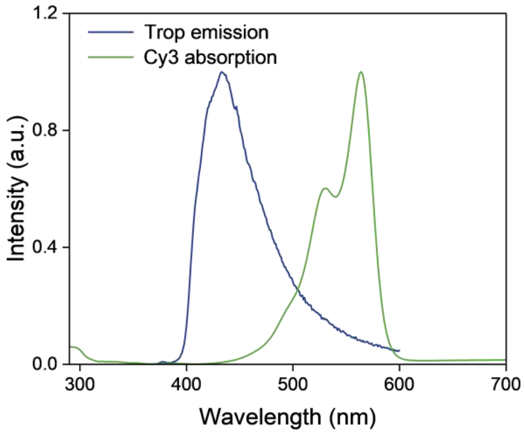

Figure S5. Normalized emission spectrum of Trop and absorption spectrum of Cy3.

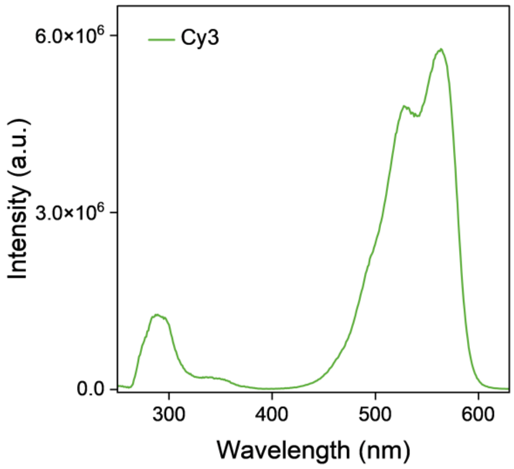

Figure S6. Excitation spectrum of Cy3 (λ_{em} = 650 nm).

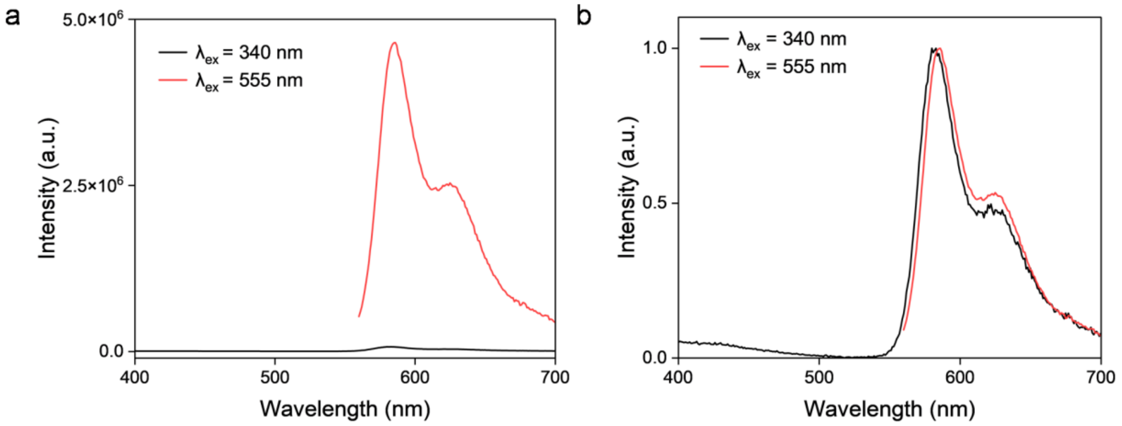

Figure S7. The original emission spectra (a) and normalized spectra (b) of Cy3 under the excitation of 340 nm and 555 nm.

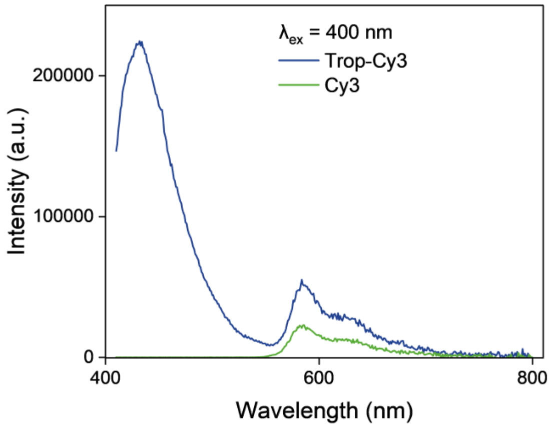

Figure S8. The emission spectra of Cy3 and Trop-Cy3 dual-dye-system under the excitation of 400 nm.

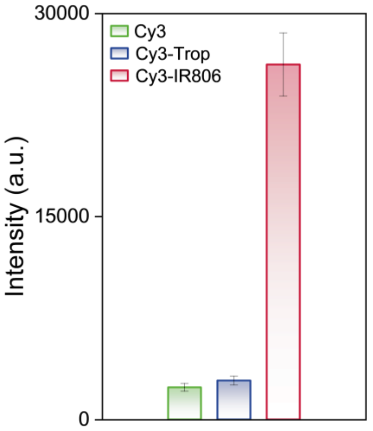

Figure S9. NIR-II emission intensity of DSNPs sensitized by single dye and double dyes under the excitation of 555 nm.

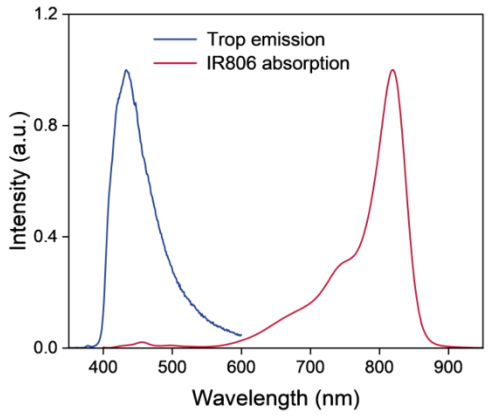

Figure S10. Normalized emission spectrum of Trop and absorption spectrum of IR806.

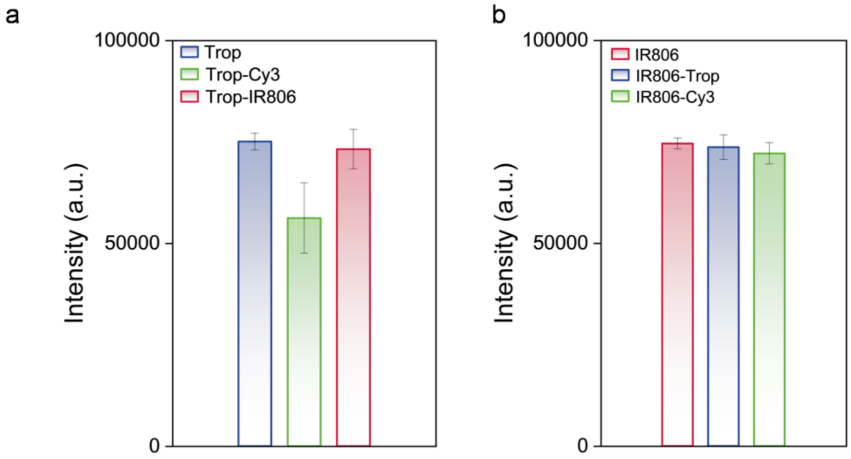

Figure S11. NIR-II emission intensity of DSNPs sensitized by single dye and double dyes under different excitation wavelengths (a. 340 nm; b. 808 nm).

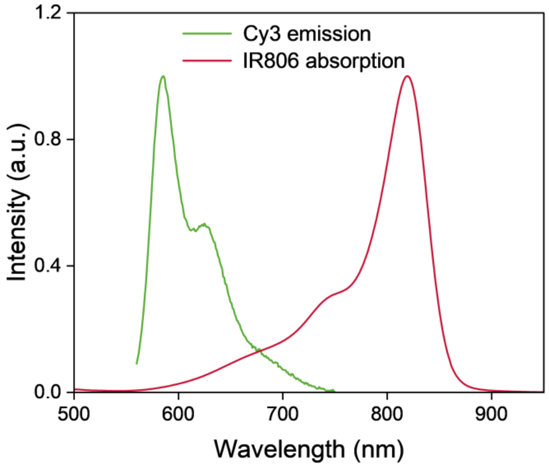

Figure S12. Normalized emission spectrum of Cy3 and absorption spectrum of IR806.

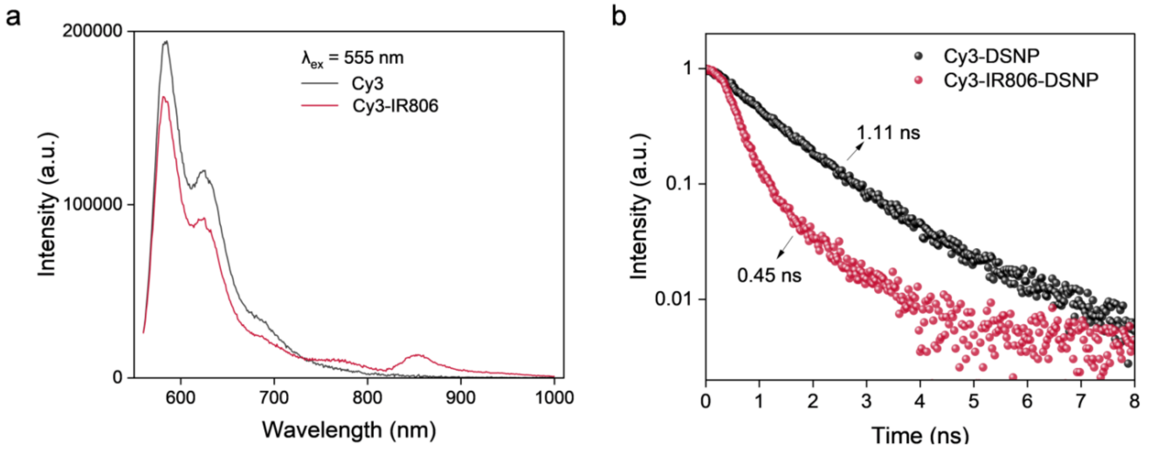

Figure S13. a. Emission spectra of Cy3 and Cy3-IR806 under the excitation of 555 nm. b. Luminescence lifetime of the Cy3-DSNPs system with and without IR806 (λ_{ex} = 510 nm, λ_{em} = 600 nm)

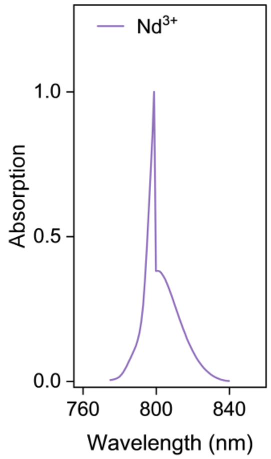

Figure S14. Normalized absorption spectrum of Nd^{3+}.

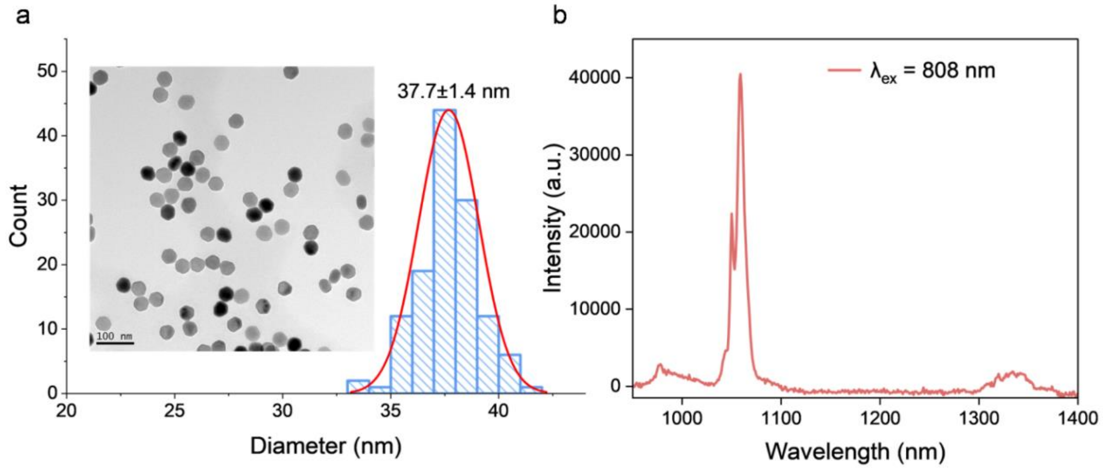

Figure S15. a. TEM image and size distribution of $NaYF_4$: 20% Nd^{3+}, with an average diameter of 37.7±1.4 nm. b. The emission spectrum of $NaYF_4$: 20% Nd^{3+} DSNPs under the excitation of 808 nm.

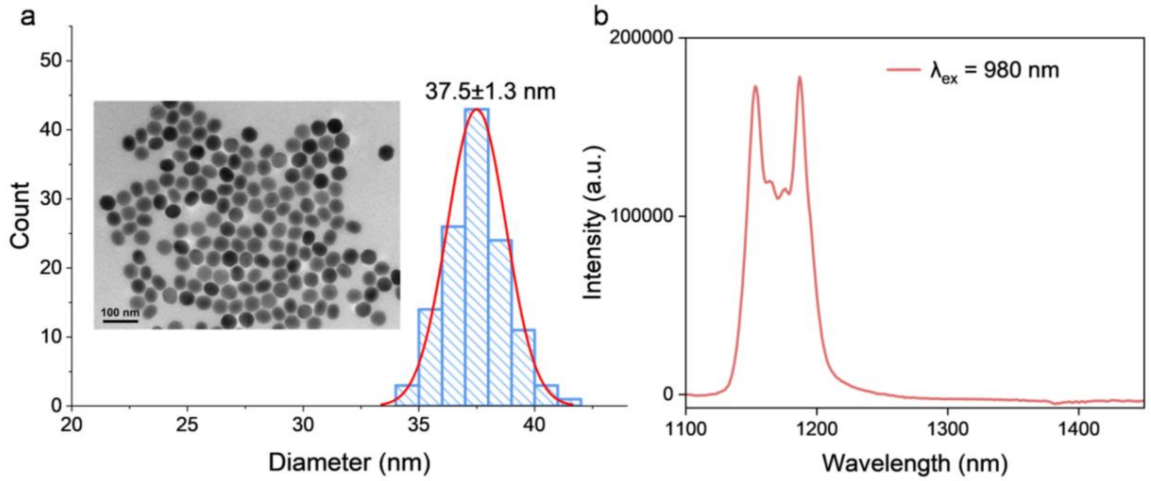

Figure S16. a. TEM image and size distribution of $NaYF_4$: 40% Yb^{3+}, 2% Ho^{3+}, with an average diameter of 37.5±1.3 nm. b. The emission spectrum of $NaYF_4$: 40% Yb^{3+}, 2% Ho^{3+} DSNPs under the excitation of 980 nm.

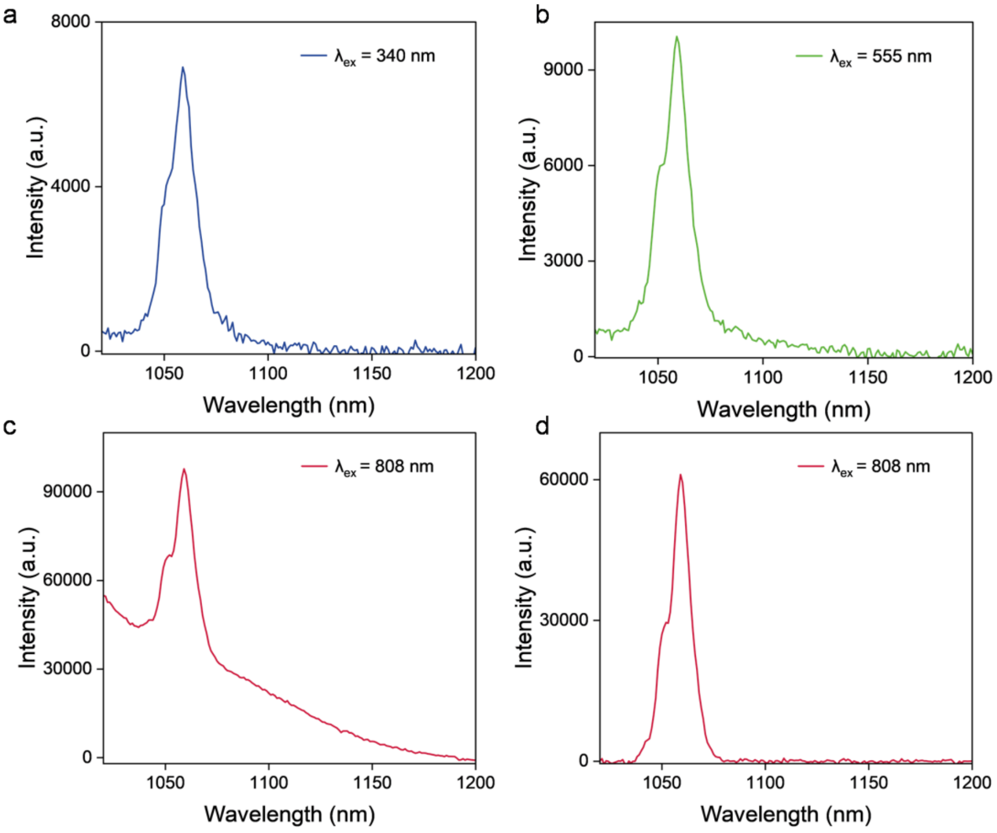

Figure S17. a-c. The emission spectra of Trop-Cy3-IR806-$NaYF_4$: 20% Nd^{3+} under the excitation of 340, 555, and 808 nm. d. The emission spectrum obtained after baseline correction under the excitation of 808 nm.

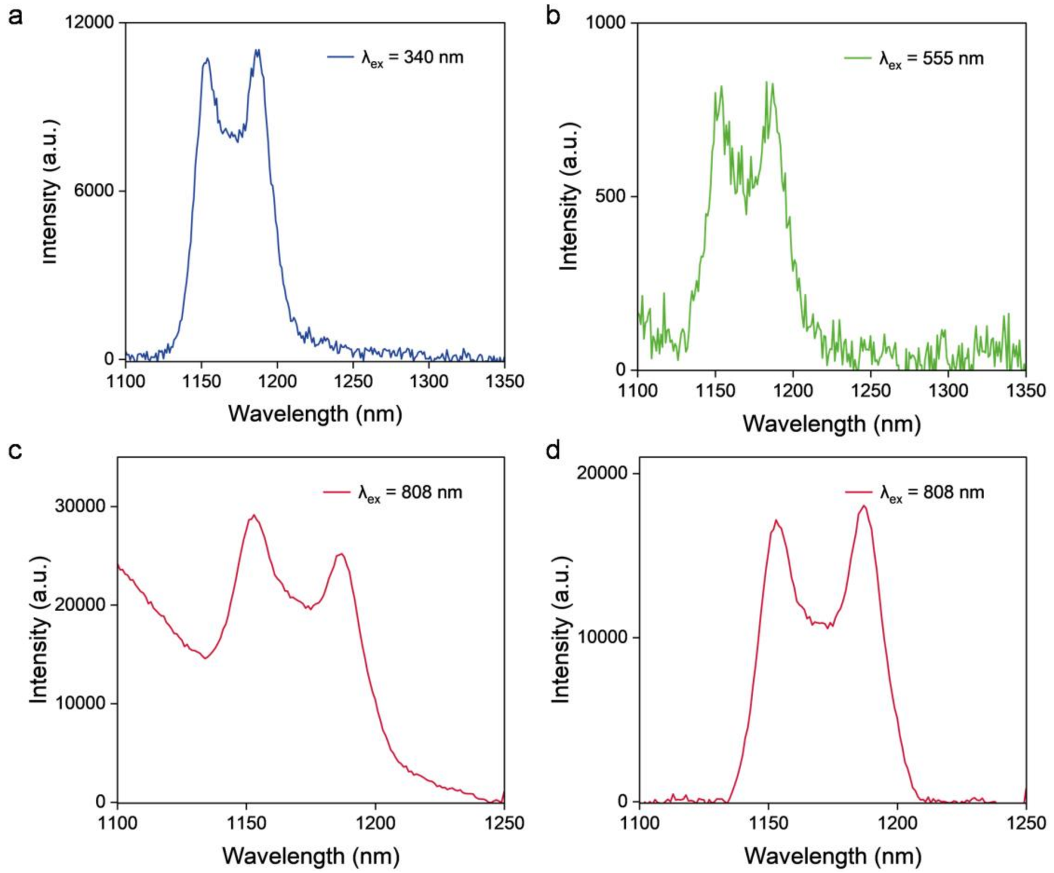

Figure S18. a-c. The emission spectra of Trop-Cy3-IR806-$NaYF_4$: 40% Yb^{3+}, 2% Ho^{3+} under the excitation of 340, 555, and 808 nm. d. The emission spectrum obtained after baseline correction under the excitation of 808 nm.

C. References

[1] C. Jiang, Y. Li, T. Jia, H. Ågren, G. Y. Chen, "Stabilizing Dye-Sensitized Upconversion Nanosystems via Singlet Oxygen Spin Flipping" *Small* **21**, (2022): e06685.

[2] Y. Liu, L. Ning, Y. Luo, Y. Huang, Z. He, H. Ma, Y. Zhao, J. Zhang, D. Liu, L. Fu, S. J. Langford, P. A. Gale, Y. Luo, G. Bao, "Stabilizing Dye-Sensitized Upconversion Hybrids by Cyclooctatetraene" *Nano Letters* **24**, (2024): 12486.

[3] G. Bao, S. Wen, W. Wang, J. Zhou, S. Zha, Y. Liu, K.-L. Wong, D. Jin, "Enhancing Hybrid Upconversion Nanosystems via Synergistic Effects of Moiety Engineered NIR Dyes" *Nano Letters* **21**, (2021): 9862.
